# Ag-Introduced Antibacterial Ability and Corrosion Resistance for Bio-Mg Alloys

**DOI:** 10.1155/2018/6023460

**Published:** 2018-07-12

**Authors:** Cijun Shuai, Yuanzhuo Zhou, Youwen Yang, Chengde Gao, Shuping Peng, Guoyong Wang

**Affiliations:** ^1^Jiangxi University of Science and Technology, Nanchang 330013, China; ^2^State Key Laboratory of High Performance Complex Manufacturing, College of Mechanical and Electrical Engineering, Central South University, Changsha 410083, China; ^3^Key Laboratory of Organ Injury, Aging and Regenerative Medicine of Hunan Province, Changsha 410008, China; ^4^The Key Laboratory of Carcinogenesis of the Chinese Ministry of Health, Xiangya Hospital, Central South University, Changsha 410078, China

## Abstract

Bone implants are expected to possess antibacterial ability and favorable biodegradability. Ag possesses broad-spectrum antibacterial effects through destroying the respiration and substance transport of bacteria. In this study, Ag was introduced into Mg-3Zn-0.5Zr (ZK30) via selective laser melting technology. Results showed that ZK30-Ag exhibited a strong and stable antibacterial activity against the bacterium Escherichia coli. Moreover, the degradation resistance was enhanced due to the comprehensive effect of positive shifted corrosion potential (from -1.64 to -1.53 V) and grains refinement. The positive shifted corrosion potential reduced the severe galvanic corrosion by lowering the corrosion potential difference between the matrix and the second phase. Meanwhile, the introduction of Ag caused the grain refinement strengthening and precipitated-phase strengthening, resulting in improved compressive yield strength and hardness. Furthermore, ZK30-0.5Ag exhibited good biocompatibility. It was suggested that Ag-modified ZK30 was potential candidate for bone implants.

## 1. Introduction

Recently, magnesium (Mg) alloy has attracted increasing attention as potential bone implant due to its favorable biocompatibility, proper mechanical properties, and inherent biodegradability [1, 2]. Mg^2+^ is an important cation in the human body and is closely related to human metabolism [3, 4]. Meanwhile, the elastic modulus of Mg alloys is close to that of natural bone, which plays an important role in avoiding stress shielding [5, 6]. Moreover, there is no need for a secondary surgery to remove the Mg bone implants due to their inherent biodegradability, which reduced the injury to patients and the cost of transplants [7]. However, the bone implants also need to possess good antibacterial property, due to the incidental bacterial infection of the implants [8, 9].

It is an effective method to endow implants with antibacterial property via introducing an antibacterial agent [9–11]. Ag is known for its broad-spectrum antibacterial effects [12, 13]. Low concentration of Ag^+^ (< 0.1 ppm) is harmless to the human body [14]. Liao et al. reported that the austenitic stainless steels with 0.3 wt.% Ag had good antibacterial property against Escherichia coli (*E. coli*) [15]. Chen et al. prepared the Ti-Ag alloys by powder metallurgy method and found that the alloys possessed good antibacterial effect against staphylococcus aureus when the Ag^+^ concentration was about 0.05 mg/L [16]. On the other hand, the degradation rate of Mg alloys in human body is too fast, because the corrosion potential of magnesium is very low (-2.364 V) [17]. The addition of Ag with a high corrosion potential of 0.799 V is expected to improve the overall corrosion potential of magnesium matrix and control its degradation rate.

In this work, Ag was introduced into Mg-3Zn-0.5Zr (ZK30) by selective laser melting technology. As a typical additive manufacturing technology, selective laser melting technology is able to fabricate Mg parts with customized shape for bone implant application [18]. Meanwhile, selective laser melting with a rapid solidification effectively reduces the grains size and composition segregation, both of which contribute to the enhancement of corrosion resistance of Mg alloys [19]. Besides, ZK30 was selected in consideration of its enough mechanical strength as well as good biocompatibility [20]. The microstructure evolution, degradation behavior, and mechanical properties were studied systematically. And the antibacterial property and antibacterial mechanism were investigated.

## 2. Materials and Methods

### 2.1. Materials

**Figure 1:**
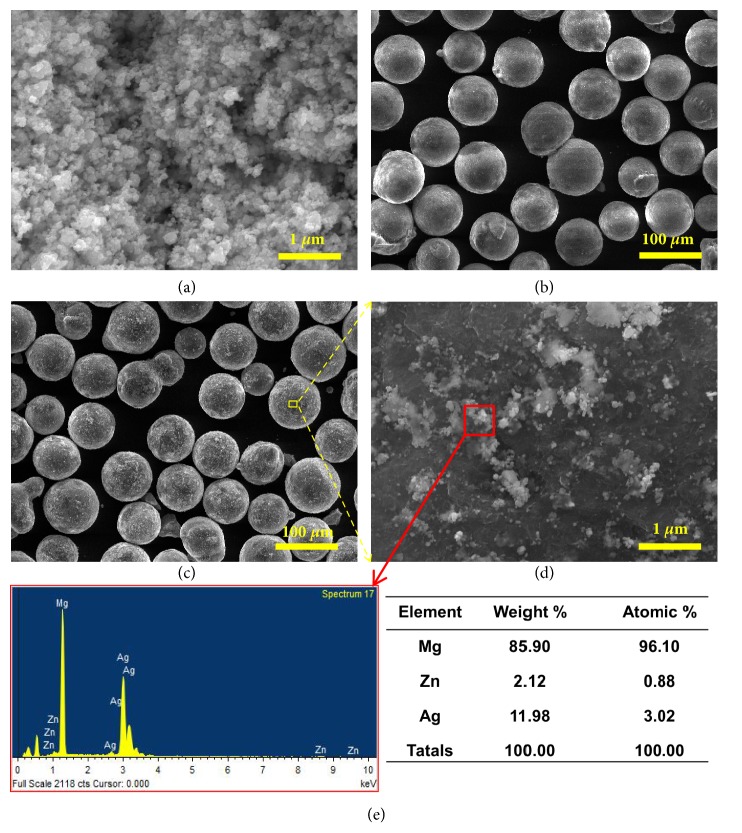
The morphologies of raw (a) Ag, (b) ZK30, and (c) mixed powders. (d) The magnified surface on mixed powders and corresponding EDS spectrum of the region marked in (d).

Ag powder with mean size of ~ 20 nm was obtained from Beijing Deke Technology Co., Ltd. (Figure 1(a)). And ZK30 powder with average size of ~ 70 *μ*m was purchased from Haoxi Nano Technology Co., Ltd. (Figure 1(b)). ZK30 and Ag powders were mixed together by ball milling for 4 hours under the gas protection of Ar. It could be observed that Ag powder was homogeneously dispersed on the surface of the ZK30 powder (Figures 1(c) and 1(d)).

### 2.2. Selective Laser Melting Process

ZK30-*x*Ag (*x*=0, 0.25, 0.5, 0.75, and 1 wt.%) samples were fabricated using a self-developed selective laser melting equipment, which was mainly composed of a control system, a fiber laser, an optical system, a motion platform, and a gas shielding device. The maximal output power of the laser was 110 W. Samples with dimensions of 10×10×5 mm^3^ were fabricated using a layer by layer method. And the processing parameters included laser power 70 W, spot size 100 *μ*m, scan speed 15 mm·s^−1^, hatch spacing 80 *μ*m, and layer thickness 100 *μ*m. Details regarding the selective laser melting process had been reported in our previous publication [21].

### 2.3. Microstructure Characterization

The prepared ZK30-*x*Ag samples were ground with SiC paper and polished with flannelette. The crystal structures of the ZK30-*x*Ag were observed using an optical microscopy after being etched with picric acid solution for 10 seconds. The microstructures were investigated utilizing a scanning electron microscope (SEM, QUANTA FEG250, FEI, USA) equipped with an energy dispersion spectrum (EDS, JSM-6490LV, JEOL, Co., Japan). Besides, the phase composition was analyzed using a X-ray diffractometer (XRD, D8-ADVANCE, Bruker AXS Inc., Germany) with a scanning rate of 8°·min^−1^ from 20° to 80°.

### 2.4. Electrochemical Tests

The electrochemical measurements were performed using an electrochemical workstation (CHI660C, CH Instrument, Shanghai, China) to obtain the open potential and polarization curves of the ZK30-*x*Ag in simulated body fluid (SBF). The electrochemical workstation contained a standard three-electrode system, including a reference electrode, an auxiliary electrode, and a working electrode. The electrochemical tests were performed in a beaker filled with the SBF. And the temperature of the solution was controlled at 37°C to reduce experimental error caused by temperature variation. Then the polarization curves were recorded with a scanning speed of 0.5 mV·s^−1^.

### 2.5. Immersion Tests

The immersion tests were carried out to detect the variations of pH value and ion concentration during immersion in SBF. The ratio of the sample surface area to solution volume was 1:150 cm^2^·mL^−1^. The pH was obtained utilizing a pH meter (Sartorius, PB-10, Germany). And the ion concentrations of Mg^2+^, Zn^2+^, and Ag^+^ were analyzed by an inductively coupled plasma atomic emission spectroscopy (ICP-AES, Perkin Elmer, Optima 5300DV, USA).

### 2.6. Mechanical Tests

The compression tests were carried out using an electronic universal testing machine (WD-D1, Shanghai Zhuoji Instruments Co., China). All the samples were compressed at a crosshead speed of 0.5 mm·min^−1^. And the hardness was measured via a HMV-2T Vickers hardness tester with a load of 9.8 N and a loading time of 15 seconds. The hardness (*HV*) was calculated by the following formula:

(1) $$\begin{array}{c} HV=0.1891\;\;F/D^{\wedge }2 \end{array}$$

where* F* was the loading force (N) and* D* was the arithmetic mean of the two indentation diagonal lengths (mm). Each sample was measured for three times.

### 2.7. Antibacterial Experiments

The antibacterial properties were evaluated via bacterial counting method using the* E. coli*. ZK30-*x*Ag samples were immersed in SBF for 72 hours to obtain extracts. And the ratio of the SBF volume to sample weight remained at 1 mL to 0.2 g. After sterilization by ultraviolet irradiation, the prepared extracts (0.1 mL) were blended with the* E. coli *suspension (0.1 mL, 1×10^6^ colony-forming units (CFU)·mL^−1^) and the SBF (0.8 mL) to get the coculture solutions, which was incubated in an incubator at 37°C for 48 hours. Afterwards, the coculture solutions (0.1 mL) were added to the nutrient agar plate and spread evenly, respectively. The broth was used as a negative control. Finally, the plates were incubated at 37°C. And the number of bacteria in each group was measured. In order to further verify the antibacterial ability of Ag, the pH values of ZK30 and ZK30-Ag extracts were both adjusted to 7.4. Then the above steps were repeated and the numbers of bacteria were obtained.

### 2.8. Cells Culture

Human osteosarcoma MG 63 cells were utilized for cells culture with the aim of investigating the biocompatibility. Dulbecco's modified eagle medium (DMEM) with 10% fetal bovine serum, 100 U·mL^−1^ penicillin, and 100 mg·mL^−1^ streptomycin was used as culture medium. ZK30-*x*Ag samples were soaked in DMEM (ratio of surface to culture medium volume 1.25 cm^2^·mL^−1^) for 3 days to obtain extracts. Then the 100% concentration extracts and 50% concentration extracts (diluted by DMEM) were prepared for subsequent cells culture.

Cells were seeded into a 96-well plate (10000 cells per mL) and incubated for 1 day. Then the culture media were replaced by 100% and 50% extracts, respectively. Meanwhile, pure DMEM was selected as control groups. After incubation for 1, 3, and 5 days, cells were incubated with 10 *μ*L CCK-8 (5 mg·mL^−1^, Sigma-Aldrich, St. Louis, MO, USA) for 2 hours. Finally, the absorbance (450 nm) was obtained using a paradigm detection platform (BECK MAN, S. Kraemer Boulevard Brea, CA).

Meanwhile, cell fluorescence staining was performed to observe the cells morphology. After culture for 1, 3, and 5 days, cells were rinsed gently with phosphate buffer saline. And then calcein-AM and ethidium homodimer-1 reagent were used to stain the cells for 15 minutes at 37°C. After rinsing gently two times using phosphate buffer saline, cells were fixed on glass slides and then observed with a fluorescence microscopy (BX60, Olympus, Japan).

### 2.9. Statistical Analysis

The data were obtained by testing three independent specimens and were expressed as means ± standard deviation. Statistical analysis of the data was carried out by the IBM SPSS software. Difference was taken to be significant as* p* value was less than 0.05.

## 3. Results and Discussion

### 3.1. Microstructures and Compositions

**Figure 2:**
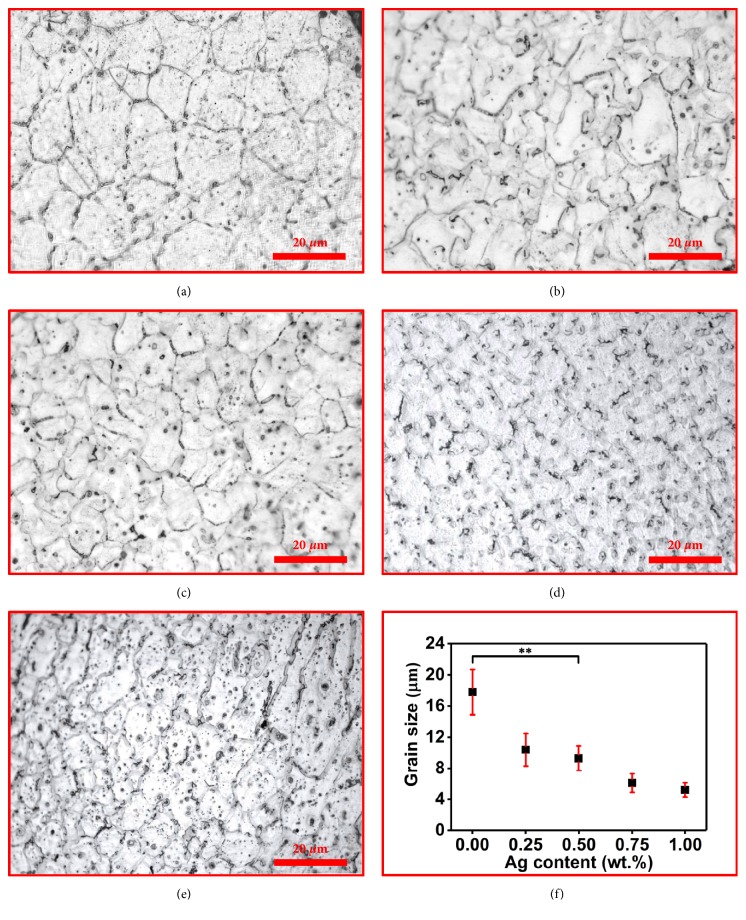
The metallographic structures of the ZK30-*x*Ag, (a) ZK30, (b) ZK30-0.25Ag, (c) ZK30-0.5Ag, (d) ZK30-0.75Ag, and (e) ZK30-1Ag, and (f) the relationship between grain size and Ag addition. Values were expressed as mean ± error,* n*=3, *∗∗p* <0.01.

The metallographic structures of the ZK30-*x*Ag were shown in Figure 2. It was obvious that the ZK30 was composed of fine equiaxed grains. For ZK30-Ag, some precipitated phases were distributed at grain boundaries or within the grains. And the grains size gradually decreased with Ag content increasing. To reveal the relationship between grains size and Ag content, the grains size was analyzed by the linear intercept method [25], with results depicted in Figure 2(f). The selective laser melting processed ZK30, ZK30-0.25Ag, ZK30-0.5Ag, ZK30-0.75Ag, and ZK30-1Ag had an average grain size of 17.8±2.9 *μ*m, 10.4±2.1*μ*m, 9.3±1.6 *μ*m, 6.1±1.2 *μ*m, and 5.2±0.9 *μ*m, respectively. It was believed that homogeneously precipitated phases at grain boundaries limited the crystal growth and promoted the grains refinement.

**Figure 3:**
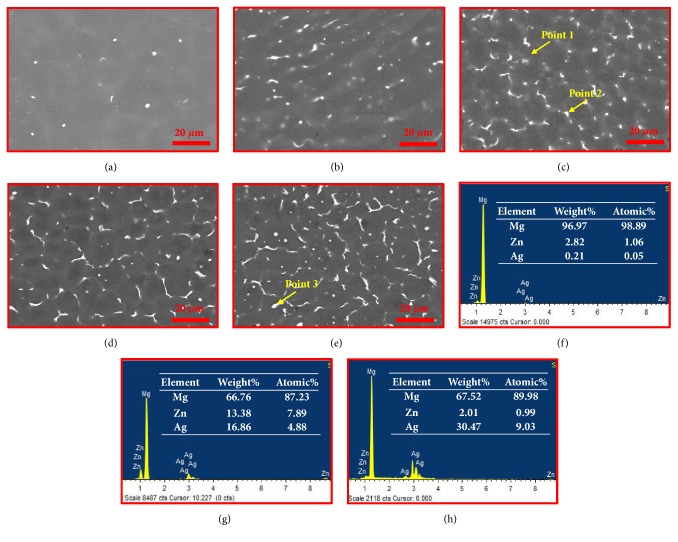
The SEM of (a) ZK30, (b) ZK30-0.25Ag, (c) ZK30-0.5Ag, (d) ZK30-0.75Ag, (e) ZK30-1Ag, and EDS results corresponding to point 1 (f), point 2 (g), and point 3 (h).

The microstructure of ZK30-*x*Ag was further investigated using SEM, with results shown in Figure 3. In the backscatter mode, distinct phases presented different grayscale levels due to distinct composition. The dark area corresponded to Mg matrix, and the bright particles corresponded to intermetallic phase which was rich in Zn or Ag. In particular, the precipitate phase, which should be Mg-Zn phase, exhibited spherical morphology in the ZK30 (Figure 3(a)). After the addition of Ag, the precipitate phases presented semicontinuous and rod-like morphologies, as shown in Figures 3(b)–3(e). And the volume fraction of the intermetallic phases gradually increased with Ag content increasing. Additionally, the EDS results revealed that 2.82 wt.% Zn and 0.21 wt.% Ag dissolved in ZK30-0.5Ag matrix, indicating a formation of supersaturated solid solution of *α*-Mg (Figure 3(f)). The precipitate phases were evidently rich in Ag (16.86 wt.%) and Zn (13.38 wt.%), as shown in Figure 3(g). Besides, the composition of second phase in ZK30-1Ag was also studied by EDS. Results showed that the second phase mainly consisted of 30.47 wt.% Ag and 67.52 wt.% Mg (Figure 3(h)), which indicated that the second phase might be Mg-Ag intermetallic.

**Figure 4:**
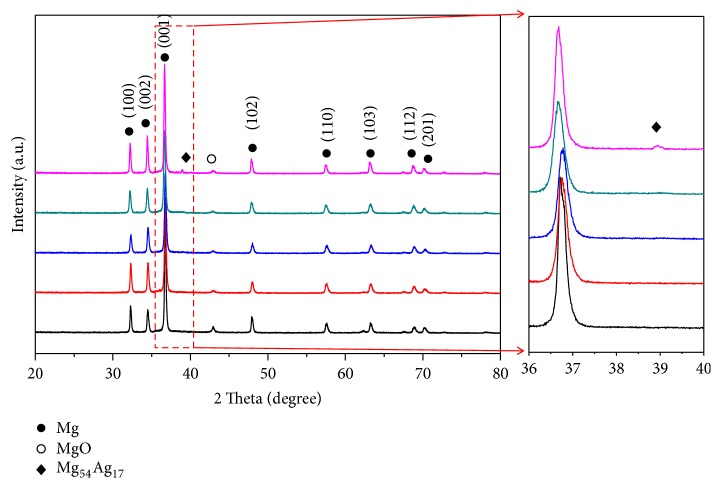
The XRD patterns of ZK30-*x*Ag with 2*θ* ranging from 20° to 80°.

The XRD patterns of selective laser melting processed ZK30-*x*Ag were obtained, as depicted in Figure 4. It could be seen that the peaks mainly corresponded to *α*-Mg phase. And no clear peaks corresponding to Zn-containing phases were detected. As previously revealed by EDS, Zn was mainly dissolved in Mg matrix. Therefore, the amount of Zn-containing phases was too small to be detected. Notably, the clear peak corresponding to Mg_54_Ag_17_ phase was detected in ZK30-1Ag. It was reasonable to deduce that the semicontinuous and rod-like precipitates at the grain boundaries of ZK30-Ag were Mg_54_Ag_17_ phase.

### 3.2. Degradation Behaviors

**Figure 5:**
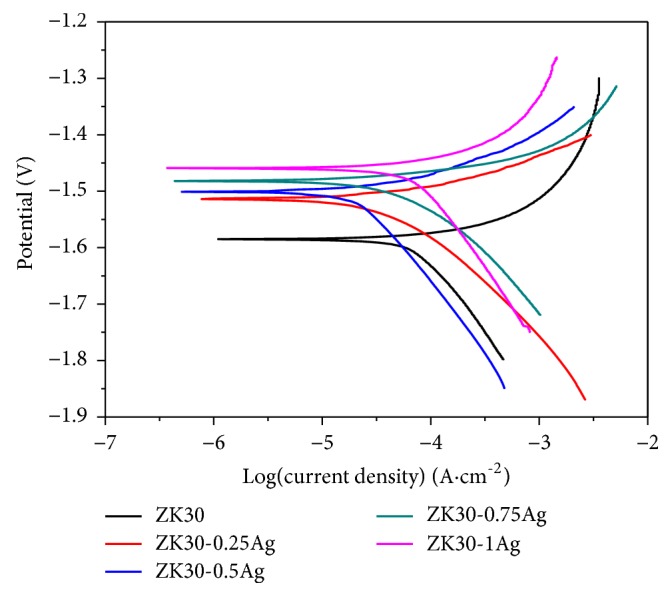
Typical electrochemical polarization curves of ZK30-xAg tested in SBF at 37°C.

The electrochemical polarization curves of ZK30-*x*Ag after immersion in SBF were depicted in Figure 5. The corresponding corrosion potential (*E*_*corr*_) and corrosion current density (*I*_*corr*_) were derived from the Tafel region, with results presented in Table 1. ZK30 presented a relative negative *E*_*corr*_ of -1.64±0.04 V. After the addition of Ag, *E*_*corr*_ shifted positively to -1.56 ~ -1.52 V. Meanwhile, *I*_*corr*_ was gradually reduced with Ag increasing to 0.5 wt.%. Specifically, ZK30-0.25Ag and ZK30-5Ag exhibited *I*_*corr*_ of 81.1±4.2 *μ*A/cm^2^ and 64.5±4.5 *μ*A/cm^2^, which was significantly lower than that of ZK30 with an *I*_*corr*_ of 109.6±4.5 *μ*A/cm^2^. It was well accepted that a positive *E*_*corr*_ represented an improved anticorrosion ability, and low *I*_*corr*_ indicated a low corrosion rate [26]. Thus, it was reasonable to infer that ZK30-0.5 possessed an improved corrosion resistance in view of its more positive *E*_*corr*_ and lowest *I*_*corr*_.

The corrosion rates of the ZK30-*x*Ag were calculated based on the electrochemical tests by the following formula [27]:

(2) $$\begin{array}{c} Corrosion\;\;rate=\frac{\left(I_{corr}Mt\right)}{n\rho F}\times 10 \end{array}$$

where* M* was the atomic weight of Mg (g),* t* equaled 3600×24×365 (s),* n* was 2 for Mg,* ρ* was the density (g·cm^−3^), and F was Faraday's constant (96485 C·mol^−1^). Results revealed that ZK30-0.5Ag exhibited a reduced corrosion rate of 1.41±0.13 mm·year^−1^, which was significantly smaller than that of ZK30 with a corrosion rate of 2.39±0.22 mm·year^−1^. Compared with some other typical biodegradable Mg alloys, such as ZK60 [22] and Mg-1Ca [23], ZK30-0.5Ag exhibited an enhanced corrosion resistance with a small degradation rate, as listed in Table 1. For extremely individual Mg alloys with particular structure, such as Mg_69_Zn_27_Ca_4_ bulk metallic glass [24], it exhibited a smaller degradation rate than ZK30-0.5Ag, which could be ascribed to the fact that the amorphous structure promoted the formation of more passive oxide film on Mg matrix.

**Figure 6:**
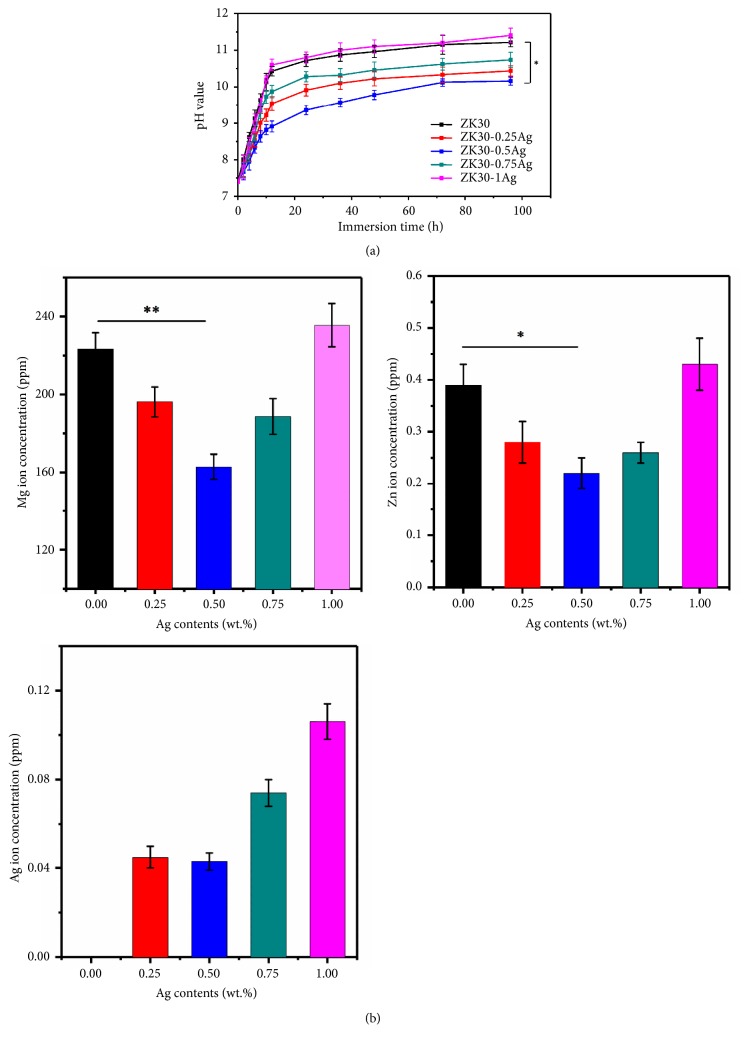
(a) The pH value of the ZK30-*x*Ag after immersion in SBF. (b) The ion concentrations of Mg^2+^, Zn^2+^, and Ag^+^ detected from the ZK30-*x*Ag extracts after immersion for 96 hours. Values were expressed as mean ± error,* n*=3, *∗p* < 0.05, and *∗∗p* <0.01.

The pH variation curves of the SBF after immersion of ZK30-*x*Ag were depicted in Figure 6(a). At the initial stage, the pH values sharply increased with immersion time extending, which was mainly due to the rapid corrosion of Mg matrix and resultant release of OH^−^ ion. After immersion for 48 hours, the pH values tended to be stable. It was believed that the corrosion products Mg(OH)_2_ covered the surface of the Mg matrix and effectively prevented the further corrosion [28]. It should be noted that after immersion for 96 hours, the extract obtained from ZK30-0.5Ag exhibited lowest pH value of 10.15±0.11, which was lower than that of ZK30 (11.21±0.12), ZK30-0.25Ag (10.43±0.14), ZK30-0.75Ag (10.33±0.21), and ZK30-1Ag (11.4±0.20), confirming its improved anticorrosion ability. The ion concentrations of Mg^2+^, Zn^2+^, and Ag^+^ after immersion for 96 hours were shown in Figure 6(b). It could be seen that both the Mg^2+^ and Zn^2+^ ion concentrations of ZK30-0.5Ag extracts were significantly lower than the other four groups, which was consistent with the results of pH tests. The Ag^+^ ion concentrations detected from the ZK30, ZK30-0.25Ag, ZK30-0.5Ag, ZK30-0.75Ag, and ZK30-1Ag extracts were 0, 0.045±0.005, 0.043±0.004, 0.074±0.006, and 0.106±0.008 ppm, respectively. Obviously, the concentration of Ag ions was within the acceptable range of human body [14].

In the present study, both the electrochemical tests and immersion tests confirmed the improved corrosion behavior of ZK30-0.5Ag. The electrochemical test revealed that *E*_*corr*_ of the Mg matrix was enhanced after the addition of Ag. It represented the fact that the surface of ZK30-*x*Ag possessed a better resistance to defend pit corrosion. The addition of Ag led to grains refinement with high density of grain boundary. And the grain boundary was one of the major crystallographic defects of Mg alloys, which acted as a corrosion barrier to inhibit the corrosion propagation [27]. On the other hand, previous researches revealed that finer grains were beneficial to the formation of a more compact corrosion product film. In fact, the molar volume ratio of the elementary unit cell of MgO to that of Mg was less than 1 [29]. Thus, the oxide film covered on Mg matrix usually exhibited cracks due the mismatch between the surface film and underlying Mg [30]. A refined microstructure could reduce the tensile stress by means of providing vacancy through high density grain boundaries, forming denser surface oxide film to defend the invasion of soaking fluid. Thus, ZK30-0.5Ag with refined microstructure exhibited reduced corrosion rate. However, the corrosion rate increased as the Ag further added to 1 wt.%. The further addition of Ag resulted in the precipitation of much intermetallic phases. The precipitated phases possessed a relative high corrosion potential and easily formed galvanic corrosion effect with neighbor Mg matrix, which in turn deteriorated the corrosion behavior.

### 3.3. Antibacterial Properties

**Figure 7:**
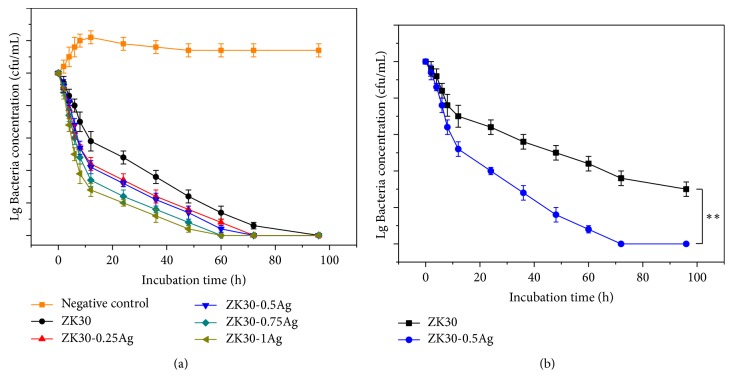
(a) The bacterial concentration of the coculture solutions after incubation at 37°C for 96 hours; (b) the bacterial concentration of the ZK30 and ZK30-0.5Ag groups after incubation in the neutral environment at 37°C for 96 hours. Values were expressed as mean ± error,* n*=3, *∗∗p* <0.01.

The bacterial concentration of the* E. coli* in the coculture solutions was measured, with results depicted in Figure 7(a). It could be seen that the number of* E. coli* in the broth culture medium firstly increased and then decreased with the prolongation of incubation time. After 48 hours of cultivation, the number of* E. coli* in the broth culture medium remained stable. In comparison, the number of* E. coli* in the ZK30-*x*Ag coculture solutions constantly decreased with culture time increasing. And after introducing Ag, the rate of colony reduction was accelerated. After cultivation for 96 hours, the bacterial concentration of the ZK30 coculture solution reduced to 0 CFU/mL, indicating that the* E. coli* was completely killed. ZK30 also exhibited a certain bactericidal effect. It was believed that the degradation of Mg alloy formed a certain alkaline environment, which might bring a certain amount of antibacterial effect [31]. In order to further verify the effect of Ag on antibacterial property, the antibacterial test of ZK30 and ZK30-0.5Ag against* E. coli* in neutral environment was carried out, with results presented in Figure 7(b). The results showed that the antibacterial ability of ZK30-0.5Ag was significantly higher than that of ZK30 in neutral environment, confirming that Ag played key roles in its improved antibacterial property.

**Figure 8:**
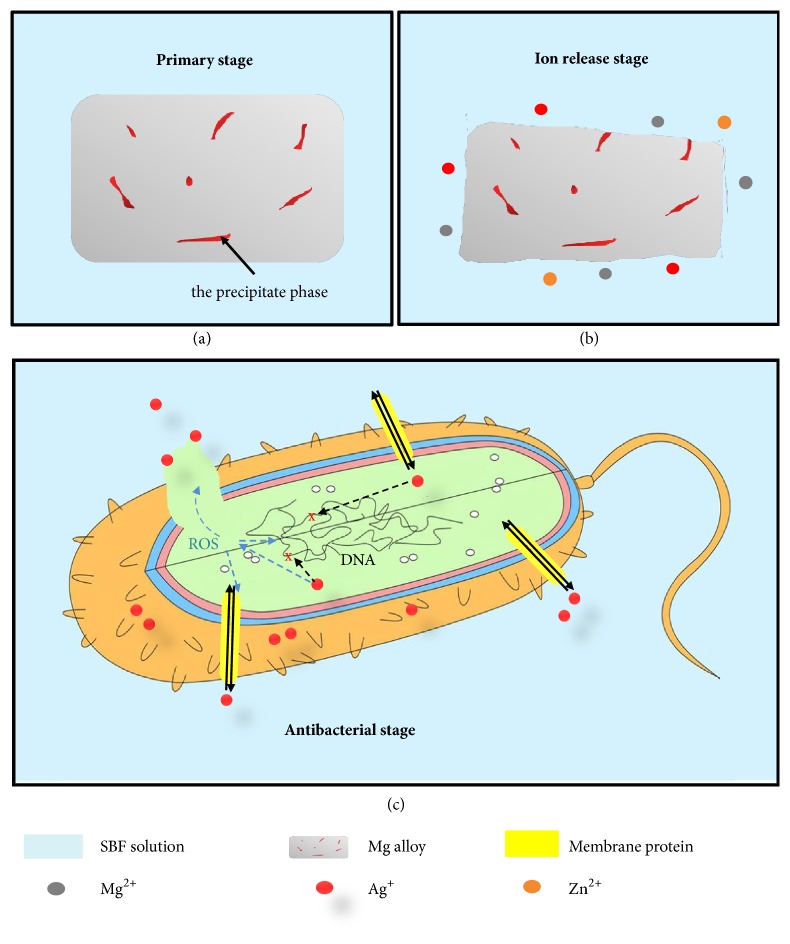
The degradation process and antibacterial mechanism of ZK30-0.5Ag.

The antibacterial mechanism of the ZK30-Ag was depicted in Figure 8. After immersion in SBF, ZK30-Ag gradually degraded and released Ag^+^ ion. The Ag^+^ ion adsorbed on the negatively charged cell membrane by electrostatic attraction and effectively penetrating the bacterial cell wall and cell membrane [32]. In addition, the Ag^+^ ion could also enter the bacteria through the surface membrane protein. Then, the Ag^+^ ion reacted with the thiamine (-SH) on the protein of the bacteria, thereby destroying their respiratory and material transport [33]. At the same time, the Ag^+^ also interfered with the synthesis of bacterial DNA, so that the bacteria lost the ability of split proliferation. Besides, the Ag^+^ might generate ROS production by direct interaction with the thiolase groups or superoxide radical scavenging enzymes in the bacteria, which could accelerate the death of the bacteria [32].

### 3.4. In Vitro Cell Response

**Figure 9:**
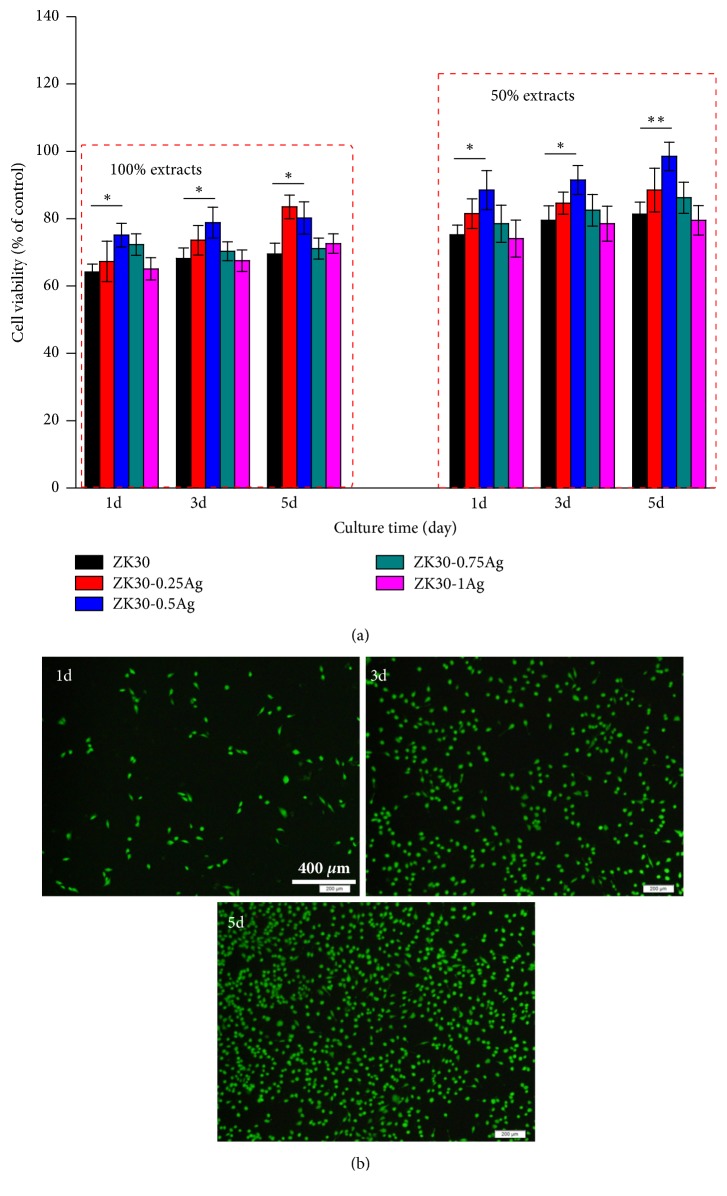
(a) The relative cell viability obtained in different extracts. Values were expressed as mean ± error,* n*=3, *∗p* < 0.05, and *∗∗p* <0.01. (b) Fluorescence images of the cells subjected to staining. The living cells were green, and the dead cells were red.

The biocompatibility of ZK30-*x*Ag was investigated using* in vitro* cell assays. And the relative cell viabilities after incubation for 1, 3, and 5 days was presented in Figure 9(a). Clearly, 100% extracts considerably reduced the cell viabilities to 64.2-75.3% after 1 day of incubation, revealing that 100% extracts had a certain inhibitory effect on cells growth. Such a negative effect was due to the local high ion concentration (OH^−^, Mg^2+^) and resultant increased osmolality pressure [34]. In general, the human body could reduce the local high ion concentration through blood circulation. To accurately mimic the human body environment, the prepared ZK30-*x*Ag extracts were diluted to 50% concentration. Results showed that 50% extracts exhibited improved cells viability ranging from 75.2 to 81.3% after 1 day of incubation. It should be noted that ZK30-0.5Ag exhibited increased cell viability compared with that obtained from ZK30, ZK30-0.25Ag, ZK30-0.75Ag, and ZK30-1Ag extracts during the whole cell culture, indicating its improved biocompatibility. It was believed that the improved corrosion resistance of ZK30-0.5Ag should be responsible for its improved biocompatibility. An improved corrosion resistance with reduced corrosion rate led to moderate ion concentration in the prepared extracts, which was more favorable for cells growth.

The morphologies of MG 63 cells cultured in 50% extract of ZK30-0.5Ag were showed in Figure 9(b). Normal cell configuration with filopodia spreading well was observed after 1, 3, and 5 days of incubation. And few dead cells were observed. With the incubation time extending, the numbers of live cells significantly increased, also confirming the good biocompatibility of ZK30-0.5Ag alloy.

### 3.5. Mechanical Properties

**Figure 10:**
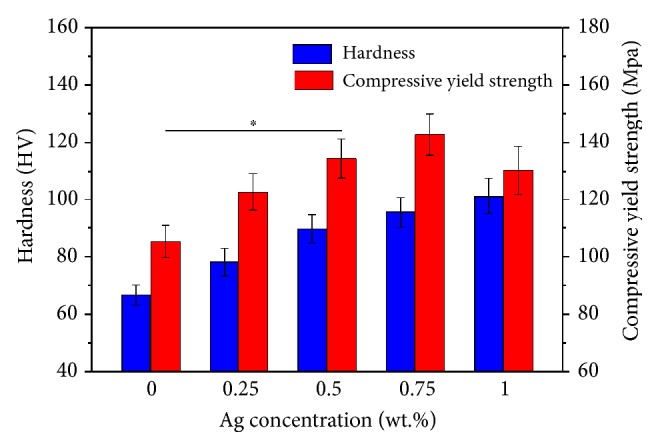
The hardness and compressive yield strength of ZK30-*x*Ag. Values were expressed as mean ± error,* n*=3, *∗p* < 0.05.

The hardness and compressive yield strength of ZK30-*x*Ag were shown in Figure 10. It could be seen that the hardness was continually enhanced with the increase of Ag. The hardness of the ZK30, ZK30-0.25Ag, ZK30-0.5Ag, ZK30-0.75Ag, and ZK30-1Ag alloy was 66.7±3.5, 78.1±4.8, 89.7±4.9, 95.5±5.4, and 101.3±6.3 HV, respectively. The enhancement of hardness was principally attributed to the grain refinement, which could be explained by the Hall-Petch relationship [35]. Additionally, the precipitate phases, which had higher hardness than that of the Mg matrix, were also conducive to the enhancement of hardness. The compressive yield strength of the ZK30-*x*Ag was obtained at room temperature. It could be seen that the compressive yield strength firstly increased and then decreased with the increase of Ag content. The compressive yield strength of the ZK30, ZK30-0.25Ag, ZK30-0.5Ag, ZK30-0.75Ag, and ZK30-1Ag alloy was 105.3±5.6, 122.7±6.5, 134.5±6.8, 142.8±7.2, and 130.4±8.3 MPa, respectively. The strains to failure were 15.3±1.2% (ZK30), 16.4±1.6% (ZK30-0.25Ag), 17.6±1.5% (ZK30-0.5Ag), 18.7±1.8% (ZK30-0.75Ag), and 15.7±2.2% (ZK30-1Ag), respectively. The enhancement of compressive yield strength was attributed to the grain refinement strengthening and precipitated-phase strengthening. However, the precipitate phases became coarse as the Ag content reached 1 wt.%, which might reduce the interface bonding strength between the *α*-Mg matrix and adjacent precipitate phase. Thus, the compressive yield strength was decreased.

## 4. Conclusions

In the present study, the microstructures evolution, antibacterial properties, degradation behaviors, and mechanical properties of ZK30-*x*Ag were systematically investigated. The grain size of ZK30-*x*Ag constantly decreased and the volume fraction of precipitate phases gradually increased with Ag increasing. ZK30-*x*Ag exhibited good antibacterial activity, revealing that Ag played a key role in the improvement of antibacterial property by destroying bacterial respiratory and material transportation. ZK30-0.5Ag exhibited enhanced degradation resistance, which was attributed to the combined effects of the grain refinement and positively shifted corrosion potential.* In vitro* cell culture revealed that ZK30-0.5Ag also had good biocompatibility. Besides, ZK30-0.5Ag possessed increased compressive yield strength and hardness as compared with ZK30, which was mainly attributed to the grain refinement strengthening and precipitated-phase strengthening. These results indicated that ZK30-0.5Ag was a promising candidate used as an antibacterial biodegradable bone implant.

## Figures and Tables

**Table 1:** Electrochemical parameters and calculated corrosion rate based on the electrochemical test. The corrosion rate of Mg_69_Zn_27_Ca_4_ bulk metallic glass was calculated based on its electrochemical corrosion current density.

Samples	*E*_*corr*_ (V)	*I*_*corr*_ (*μ*A/cm^2^)	Corrosion rate (mm/year)
ZK30	-1.64±0.04	109.6±4.5	2.39±0.22
ZK30-0.25Ag	-1.52±0.05	81.1±4.2	1.77±0.15
ZK30-0.5Ag	-1.53±0.03	64.5±4.5	1.41±0.13
ZK30-0.75Ag	-1.54±0.02	74.3±3.5	1.62±0.16
ZK30-1Ag	-1.56±0.03	120.23±6.7	2.62±0.25
Mg–1Ca [22]	-1.89	76.14	1.74
ZK60 [23]	-1.61	126.04	2.88
Mg_69_Zn_27_Ca_4_[24]	-1.12	1.55	0.04

## Data Availability

The data used to support the findings of this study are available from the corresponding author upon request.

## References

[1] Zhao D., Witte F., Lu F., Wang J., Li J., Qin L. (2017). Current status on clinical applications of magnesium-based orthopaedic implants: A review from clinical translational perspective. *Biomaterials*.

[2] Zhao D., Huang S., Lu F. (2016). Vascularized bone grafting fixed by biodegradable magnesium screw for treating osteonecrosis of the femoral head. *Biomaterials*.

[3] Jin W., Wu G., Feng H., Wang W., Zhang X., Chu P. K. (2015). Improvement of corrosion resistance and biocompatibility of rare-earth WE43 magnesium alloy by neodymium self-ion implantation. *Corrosion Science*.

[4] Guo E., Phillion A. B., Cai B. (2017). Dendritic evolution during coarsening of Mg-Zn alloys via 4D synchrotron tomography. *Acta Materialia*.

[5] Staiger M. P., Pietak A. M., Huadmai J., Dias G. (2006). Magnesium and its alloys as orthopedic biomaterials: a review. *Biomaterials*.

[6] Hong D., Saha P., Chou D.-T. (2013). In vitro degradation and cytotoxicity response of Mg-4% Zn-0.5% Zr (ZK40) alloy as a potential biodegradable material. *Acta Biomaterialia*.

[7] Li Y., Liu L., Wan P. (2016). Biodegradable Mg-Cu alloy implants with antibacterial activity for the treatment of osteomyelitis: In vitro and in vivo evaluations. *Biomaterials*.

[8] Guimond-Lischer S., Ren Q., Braissant O., Gruner P., Wampfler B., Maniura-Weber K. (2016). Vacuum plasma sprayed coatings using ionic silver doped hydroxyapatite powder to prevent bacterial infection of bone implants. *Biointerphases*.

[9] Zhao Y., Jamesh M. I., Li W. K. (2014). Enhanced antimicrobial properties, cytocompatibility, and corrosion resistance of plasma-modified biodegradable magnesium alloys. *Acta Biomaterialia*.

[10] Zheng Y. F., Zhang B. B., Wang B. L. (2011). Introduction of antibacterial function into biomedical TiNi shape memory alloy by the addition of element Ag. *Acta Biomaterialia*.

[11] Li H. F., Qiu K. J., Zhou F. Y., Li L., Zheng Y. F. (2016). Design and development of novel antibacterial Ti-Ni-Cu shape memory alloys for biomedical application. *Scientific Reports*.

[12] He M., Wang Q., Wang R., Xie Y., Zhao W., Zhao C. (2017). Design of Antibacterial Poly(ether sulfone) Membranes via Covalently Attaching Hydrogel Thin Layers Loaded with Ag Nanoparticles. *ACS Applied Materials & Interfaces*.

[13] Badea M., Braic M., Kiss A. (2016). Influence of Ag content on the antibacterial properties of SiC doped hydroxyapatite coatings. *Ceramics International*.

[14] Zheng H., Yan M., Fan X.-X. (2012). A heptamethine cyanine-based colorimetric and ratiometric fluorescent chemosensor for the selective detection of Ag + in an aqueous medium. *Chemical Communications*.

[15] Tang G. H., Liu K., Cao H. J. (2010). Effect of silver on antibacterial properties of stainless steel. *Applied Surface Science*.

[16] Chen M., Zhang E., Zhang L. (2016). Microstructure, mechanical properties, bio-corrosion properties and antibacterial properties of Ti-Ag sintered alloys. *Materials Science and Engineering C: Materials for Biological Applications*.

[17] Li J., Jiang Q., Sun H., Li Y. (2016). Effect of heat treatment on corrosion behavior of AZ63 magnesium alloy in 3.5 wt.% sodium chloride solution. *Corrosion Science*.

[18] Gu D. D., Meiners W., Wissenbach K., Poprawe R. (2012). Laser additive manufacturing of metallic components: Materials, processes and mechanisms. *International Materials Reviews*.

[19] Deng Y., Yang Y., Gao C. (2018). Mechanism for corrosion protection of *β*-TCP reinforced ZK60 via laser rapid solidification. *International Journal of Bioprinting*.

[20] Huan Z. G., Leeflang M. A., Zhou J., Fratila-Apachitei L. E., Duszczyk J. (2010). In vitro degradation behavior and cytocompatibility of Mg-Zn-Zr alloys. *Journal of Materials Science: Materials in Medicine*.

[21] Yang Y., Wu P., Lin X. (2016). System development, formability quality and microstructure evolution of selective laser-melted magnesium. *Virtual and Physical Prototyping*.

[22] Li Z., Gu X., Lou S., Zheng Y. (2008). The development of binary Mg-Ca alloys for use as biodegradable materials within bone. *Biomaterials*.

[23] Lin D.-J., Hung F.-Y., Yeh M.-L., Lui T.-S. (2015). Microstructure-modified biodegradable magnesium alloy for promoting cytocompatibility and wound healing in vitro. *Journal of Materials Science: Materials in Medicine*.

[24] Guo S. F., Chan K. C., Jiang X. Q. (2013). Atmospheric RE-free Mg-based bulk metallic glass with high bio-corrosion resistance. *Journal of Non-Crystalline Solids*.

[25] Misra R. D. K., Challa V. S. A., Venkatsurya P. K. C., Shen Y. F., Somani M. C., Karjalainen L. P. (2015). Interplay between grain structure, deformation mechanisms and austenite stability in phase-reversion-induced nanograined/ultrafine-grained austenitic ferrous alloy. *Acta Materialia*.

[26] Yang Y., Guo X., He C., Gao C., Shuai C. (2018). Regulating Degradation Behavior by Incorporating Mesoporous Silica for Mg Bone Implants. *ACS Biomaterials Science & Engineering*.

[27] Zhang H. J., Zhang D. F., Ma C. H., Guo S. F. (2013). Improving mechanical properties and corrosion resistance of Mg-6Zn-Mn magnesium alloy by rapid solidification. *Materials Letters*.

[28] Gao C., Peng S., Feng P., Shuai C. (2017). Bone biomaterials and interactions with stem cells. *Bone Research*.

[29] Shuai C., Yang Y., Wu P. (2017). Laser rapid solidification improves corrosion behavior of Mg-Zn-Zr alloy. *Journal of Alloys and Compounds*.

[30] Orlov D., Ralston K. D., Birbilis N., Estrin Y. (2011). Enhanced corrosion resistance of Mg alloy ZK60 after processing by integrated extrusion and equal channel angular pressing. *Acta Materialia*.

[31] Hou P., Zhao C., Cheng P. (2017). Reduced antibacterial property of metallic magnesium. *Biomedical Materials*.

[32] Amin Yavari S., Loozen L., Paganelli F. L. (2016). Antibacterial Behavior of Additively Manufactured Porous Titanium with Nanotubular Surfaces Releasing Silver Ions. *ACS Applied Materials & Interfaces*.

[33] Marambio-Jones C., Hoek E. M. V. (2010). A review of the antibacterial effects of silver nanomaterials and potential implications for human health and the environment. *Journal of Nanoparticle Research*.

[34] Fischer J., Prosenc M. H., Wolff M., Hort N., Willumeit R., Feyerabend F. (2010). Interference of magnesium corrosion with tetrazolium-based cytotoxicity assays. *Acta Biomaterialia*.

[35] Xu N., Shen J., Xie W., Wang L., Wang D., Min D. (2010). Abnormal distribution of microhardness in tungsten inert gas arc butt-welded AZ61 magnesium alloy plates. *Materials Characterization*.

